# The London and Counties Medical Protection Society, Limited

**Published:** 1918-06-01

**Authors:** 


### THE LONDON AND COUNTIES MEDICAL PROTECTION SOCIETY, LIMITED.

The annual report of this Society has recently been distributed amongst its members. Owing to the high cost of paper and printing and the shortage of labour, the Council of the Society has decided, this year as last year, to issue only a brief report to distribute it amongst its members only, and not to all the members of the medical and dental professions as was its former practice. The number of members is practically the same as last year. Some of those who have joined the naval and military Forces have resigned their membership. This is a short-sighted policy. Under the novel conditions of their work there are bound to arise occasions when the advice and support of a Medical Defence Society will be of the greatest value to them. The experience of the Society since the war began proves this. The amount of litigation during the past year has been below the average, but recently there has been a slight increase, especially in libel actions. The Society has been able to prevent legal action in many cases of alleged negligence by discussing matters freely and in a conciliatory spirit with the complainants. This is one of the most valuable aspects of the Society's work, and a well-merited compliment is paid to Dr. Hugh Woods, the general secretary, for the tact and patience he has shown in conducting negotiations. The Society was successful in establishing the claims of many of its members to substantial sums due to them under contract with the military authorities for examining recruits. It failed only when the member had accepted reduced amounts and given receipts in full discharge.

When a dispute arises medical men should take no steps except under legal advice. Many a man has failed to establish a perfectly just and legal claim because he has non-suited himself by action taken without appreciation of the legal consequences. As in previous years the administration of the National Health Insurance Act has given rise to many applications for help and protection. The defects in the Act to which the Society specially draws attention are the method of calculating the sums due to doctors for their services, the arbitrary surcharging of doctors when the cost of drugs prescribed by them is regarded as too high, and the worrying of doctors by elaborate inquiries into petty complaints by patients. The Society insures its members, for an additional subscription of ten shillings a year, against any pecuniary loss from litigation, when the defence is undertaken by the Society, up to an amount which is not likely to be exceeded. The financial position of the Society is a strong one. Its income and expenditure account shows a balance of £2,086. It has accumulated large reserve funds and has written down its investments to their market values on December 31, 1917. Apart from its work in defending and advising its members, the Society has successfully prosecuted several unqualified practitioners and has given important evidence before the Departmental Committee appointed to consider the working of the Dentists Act.

